# Validation and comparison of two NGS assays for the detection of EGFR T790M resistance mutation in liquid biopsies of NSCLC patients

**DOI:** 10.18632/oncotarget.24908

**Published:** 2018-04-06

**Authors:** Claudia Vollbrecht, Annika Lehmann, Dido Lenze, Michael Hummel

**Affiliations:** ^1^ Charité-Universitätsmedizin Berlin Corporate Member of Freie Universität Berlin, Humboldt-Universität zu Berlin, and Berlin Institute of Health, Institute of Pathology, Berlin, Germany; ^2^ German Cancer Consortium (DKTK), Partner Site Berlin, Berlin, Germany; ^3^ German Cancer Research Center (DKFZ), Heidelberg, Germany

**Keywords:** liquid biopsy, cfDNA, NSCLC, T790M, NGS

## Abstract

Analysis of circulating cell-free DNA (cfDNA) derived from peripheral blood (“liquid biopsy”) is an attractive alternative to identify non-small cell lung cancer (NSCLC) patients with the *EGFR* T790M mutation eligible for 3rd generation tyrosine kinase inhibitor therapy.

We evaluated two PCR-based next generation sequencing (NGS) approaches, one including unique molecular identifiers (UMI), with focus on highly sensitive *EGFR* T790M mutation detection. Therefore, we extracted and sequenced cfDNA from synthetic plasma samples spiked with mutated DNA at decreasing allele frequencies and from 21 diagnostic NSCLC patients. Data evaluation was performed to determine the limit of detection (LoD), accuracy, specificity and sensitivity of both assays.

Considering all tested reference dilutions and mutations the UMI assay performed best in terms of LoD (1% vs. 5%), sensitivity (95.8% vs. 81.3%), specificity (100% vs. 93.8%) and accuracy (96.9% vs. 84.4%). Comparing mutation status of diagnostic samples with both assays showed 81.3% concordance with primary mutation verifiable in 52% of cases. *EGFR* T790M was detected concordantly in 6/7 patients with allele frequencies from 0.1% to 27%. In one patient, the T790M mutation was exclusively detectable with the UMI assay.

Our data demonstrate that both assays are applicable as multi-biomarker NGS tools enabling the simultaneous detection of primary *EGFR* driver and resistance mutations. However, for mutations with low allelic frequencies the use of NGS panels with UMI facilitates a more sensitive and reliable detection.

## INTRODUCTION

The detection of activating epidermal growth factor receptor (*EGFR*) mutations in a subset of non-small cell lung cancer (NSCLC) patients and the development of corresponding tyrosine kinase inhibitors (TKI) has led to an important expansion of the therapeutic options and to a significant improvement of the clinical outcome of these patients [1, 2]. However, despite an initial and usually dramatic treatment response to TKI therapy patients inevitably acquire resistance to TKI treatment [3–5]. Several mechanisms for the development of acquired resistance have been reported of which the most frequent one is a secondary *EGFR* mutation at amino acid position 790 (T790M) [6, 7]. To overcome T790M mediated resistance, third generation TKI (e.g. osimertinib) have been developed that irreversibly bind to the mutated EGF receptor providing a further therapeutic option after treatment with TKI targeting activating *EGFR* mutations [8–10]. In order to identify patients eligible for treatment with third generation TKI, an early detection of the T790M resistance mutation is mandatory. Currently, tumor tissue is usually employed for the identification of activating as well as T790M resistance *EGFR* mutations. The analysis of tissue samples is considered as gold standard since it provides combined information about tumor histology, tumor content and genomic alterations. Nevertheless, the use of tissue and biopsies in particular also has several shortcomings. They represent only a snapshot of the entire tumor and thus cannot capture its entire genomic heterogeneity. Furthermore, in the metastatic situation different (actionable) molecular alterations can develop at different sites, which will not be depicted by a single tissue sample usually taken from the best accessible location. In addition, thoracic biopsies show considerable high rates of clinical complications and can be technically challenging or even impossible or clinically not feasible [11, 12].

In contrast, liquid biopsies (LB) can be obtained by minimal invasive blood draws and are therefore accessible at almost all clinical situations. They have been shown to contain tumor genomic information in which circulating cell-free tumor DNA (ctDNA) appears to be the most stable and most easily accessible source [13, 14]. Therefore, the use of LB allows the characterization of somatic mutations at baseline and dynamically during treatment, enabling the early detection of resistance mutations as well as monitoring of therapy response and minimal residual disease [11, 15]. For these reasons the analysis of LB has become a promising tool to verify the T790M mutation in NSCLC patients, which developed a resistance to TKI therapy against activating *EGFR* mutations [16, 17]. Nevertheless, the analysis of LB is also challenging due to the mainly low amounts of circulating cell-free DNA (cfDNA) and the very low representation of mutated cfDNA molecules extractable from the blood plasma. Therefore, methods which are able to detect small number of mutated molecules in an abundance of unmutated DNA fragments with high sensitivity and specificity are required [18, 19]. Currently quantitative PCR, amplification refractory mutation system (ARMS), digital PCR, beads, emulsion, amplification, and magnetics (BEAMing) and next generation sequencing (NGS) are widely-used [15]. While these methods enable a sensitive detection down to 0.01%, only NGS facilitates the parallel detection of a broader range of mutations by multi-gene or multi-target gene panels. To overcome the drawbacks of PCR-based NGS (e.g. DNA polymerase errors, generation of PCR duplicates as a result of sequencing the same molecule multiple times, etc.), the addition of unique molecular identifiers (UMI), random nucleotide sequences barcoding each DNA-fragment prior to PCR amplification, was introduced [20]. The UMI allow to distinguish reads amplified from the same original DNA molecule based on identical UMI and to identify molecules containing true variants. To compare PCR-based gene-panels with and without UMI for the detection of T790M mutations in LB, we tested two commercially available NGS panels. Synthetic reference plasma samples as well as diagnostic samples from routine setting were included in our study.

## RESULTS

### Results from reference samples

Extraction from synthetic plasma yielded about 400 ng total cfDNA (mean: 432 ng, range: 364 ng–480 ng), which is in accordance with the specification of the vendor. CLv2 panel sequencing resulted in mean 21,133 average reads per amplicon (range 17,514–24,438) (Supplementary Table 3). For the OLcfDNA assay the median read coverage (median coverage across the targets) for 0.1% limit of detection (LoD) is specified as > 25,000 with a median molecular coverage (median number of individual interrogated DNA molecules across the targets) of > 2,500. These specifications were achieved for each sample except one 1% allele frequency reference, which showed slightly less median molecular coverage.

The lowest detectable allele frequency for all eight tested mutations was 5% for CLv2 and 1% for OLcfDNA assay (Table 1). In respect to mutation type, LoD for point mutation confirmation was 1% for both assays. At 0.1% allele frequency 92% of point mutations were still detectable with OLcfDNA, in contrast to 67% with CLv2 assay. For insertions/deletions (indels), 0.1% allele frequency variants were not detectable with CLv2 but in 75% of samples with the OLcfDNA assay. *EGFR* T790M resistance mutation was detectable to 0.1% allele frequency with both assays.

**Table 1:** Limit of detection (LoD) for CLv2 and OLcfDNA panel determined with reference standard DNA extracted from synthetic plasma samples

	AF Reference cfDNA	LoD	
		CLv2	OLcfDNA
**All 8 hotspot mutations**	**5%**	16/16	16/16
		(100%)	(100%)
	**1%**	14/16	16/16
		(88%)	(100%)
	**0.1%**	8/16	14/16
		(50%)	(88%)
**Point mutations**	**5%**	12/12	12/12
		(100%)	(100%)
	**1%**	12/12	12/12
		(100%)	(100%)
	**0.1%**	8/12	11/12
		(67%)	(92%)
**Insertions/Deletions**	**5%**	4/4	4/4
		(100%)	(100%)
	**1%**	3/4	4/4
		(75%)	(100%)
	**0.1%**	0/4	3/4
		(0%)	(75%)
***EGFR* T790M**	**5%**	2/2	2/2
		(100%)	(100%)
	**1%**	2/2	2/2
		(100%)	(100%)
	**0.1%**	2/2	2/2
		(100%)	(100%)

AF: Allele Frequency; LoD: Limit of Detection.

Considering all tested reference dilutions (5%, 1%, 0.1% and wildtype) CLv2 panel showed 100% specificity for indel detection and 92.3% for point mutation analysis (Table 2). Sensitivity of point mutation detection was significantly higher than for indel detection (88.9% vs. 58.3%). Deviations to expected mutation status were higher for indel detection than for point mutations (68.8% vs 89.9% accuracy). The OLcfDNA assay reached 96.9% accuracy, 100% specificity and > 95% sensitivity, whereas sensitivity of indel detection was slightly lower than for point mutation analysis (91.7% vs. 97.2%). Sensitivity for 0.1% allele frequency mutation detection was 87.5%, with indel detection showing again a decreased sensitivity compared to point mutation analysis (91.7% vs 75.0%) (Table 3).

**Table 2:** Sensitivity, specificity, and accuracy for CLv2 and OLcfDNA panel determined with reference standard DNA extracted from synthetic plasma samples

		Results including all reference dilutions (5% AF, 1% AF, 0.1% AF and wildtype)	
		CLv2	OLcfDNA
**All 8 hotspot mutations**	**Sensitivity**	81.3%	95.8%
	**Specificity**	93.8%	100%
	**PPV**	97.5%	100%
	**NPV**	62.5%	88.9%
	**Accuracy**	84.4%	96.9%
**Point mutations**	**Sensitivity**	88.9%	97.2%
	**Specificity**	92.3%	100%
	**PPV**	97.0%	100%
	**NPV**	75.0%	92.3%
	**Accuracy**	89.8%	97.9%
**Insertions/Deletions**	**Sensitivity**	58.3%	91.7%
	**Specificity**	100%	100%
	**PPV**	100%	100%
	**NPV**	44.4%	80.0%
	**Accuracy**	68.8%	93.8%

AF: Allele Frequency; PPV: positive predictive value; NPV: negative predictive value.

**Table 3:** Sensitivity, specificity and accuracy for CLv2 and OLcfDNA panel determined with 0.1% allele frequency mutated reference standard DNA extracted from synthetic plasma samples

		Results for 0.1% allele frequency reference dilution	
		CLv2	OLcfDNA
**All 8 hotspot mutations**	**Sensitivity**	50%	87.5%
	**Specificity**	93.8%	100%
	**PPV**	88.9%	100%
	**NPV**	65.2%	88.9%
	**Accuracy**	71.9%	93.8%
**Point mutations**	**Sensitivity**	66.7%	91.7%
	**Specificity**	91.7%	100%
	**PPV**	88.9%	100%
	**NPV**	73.3%	92.3%
	**Accuracy**	79.2%	95.8%
**Insertions/Deletions**	**Sensitivity**	0%	75.0%
	**Specificity**	100%	100%
	**PPV**	NA	100%
	**NPV**	50.0%	80.0%
	**Accuracy**	50.0%	87.5%

PPV: positive predictive value; NPV: negative predictive value; NA: not analyzable.

**Figure 1:**
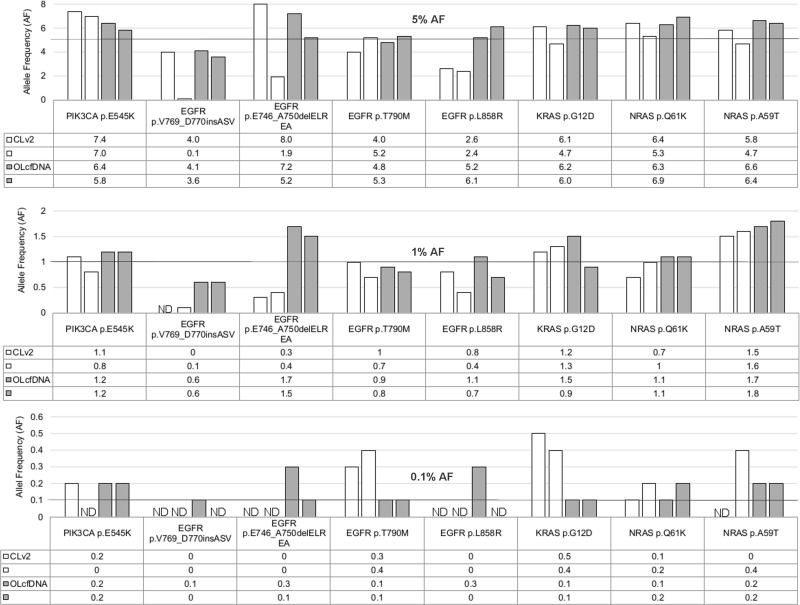
Colon Lung version 2 panel (CLv2, white bars) and Oncomine Lung cf DNA assay (OLcfDNA, grey bars) detected allele frequencies (AF) for 5%, 1% and 0.1% reference mutations; 5% AF reference mutations were detected with both assays done by double determination CLv2 assay showed a drop out for the 1% *EGFR* insertion in one of the duplicates. All other 1% reference mutations were detected with both assays. 0.1% *EGFR* insertion, *EGFR* deletion and *EGFR* L858R point mutation references were not detected and *PIK3CA* as well as *NRAS* reference mutation showed a drop out for one of the duplicate samples with CLv2 assay. All 0.1% reference mutations, except one of the duplicate samples for *EGFR* insertion and *EGFR* L858R point mutation, were detected. AF: allele frequency; ND: Not Detected.

**Figure 2:**
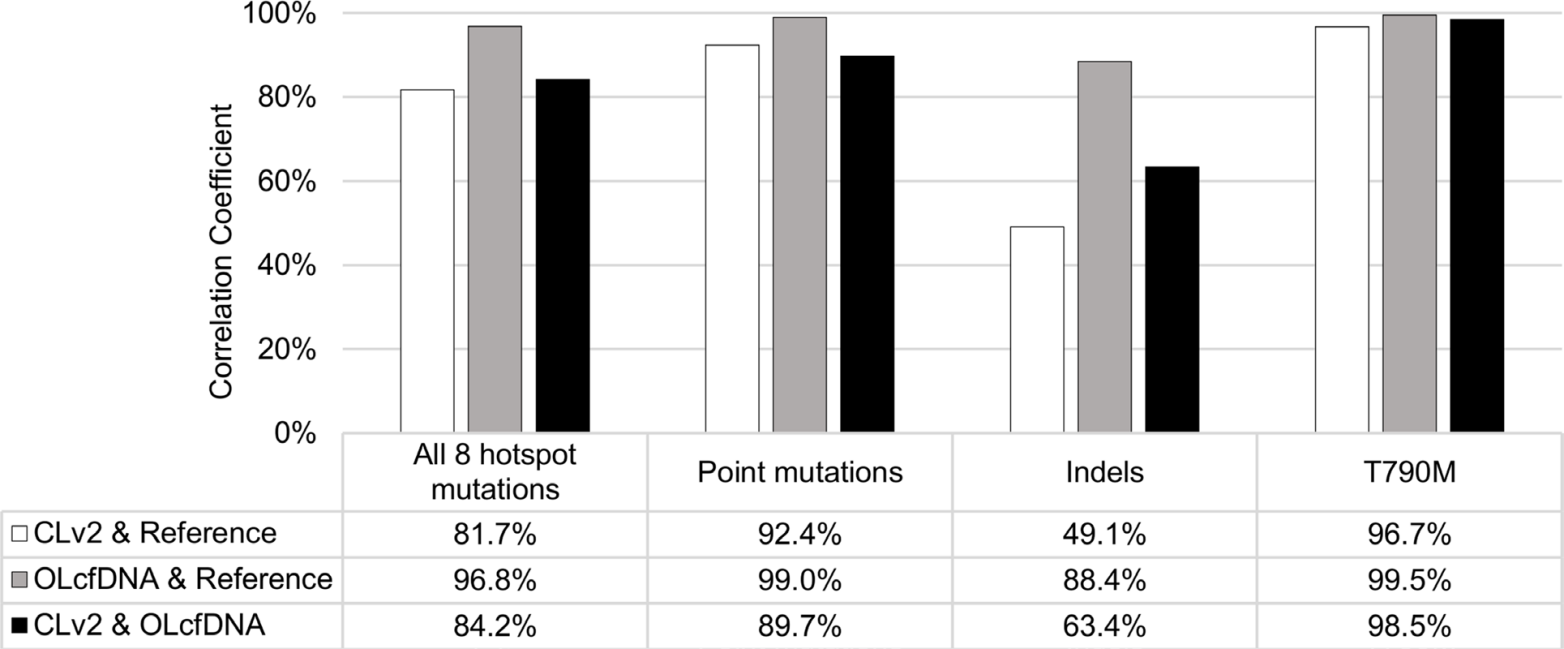
Correlation of expected and detected mutation allele frequencies of the reference samples for each panel (white and grey bars) and between both panels (Colon Lung version 2 (CLv2) and Oncomine Lung cfDNA (OLcfDNA), black bars); The correlation between the expected and detected allele frequencies of the reference samples was higher with OLcfDNA than with CLv2 panel Both assays achieved the highest correlation of expected and detected allele frequencies for *EGFR* T790M mutation (99.5% OLcfDNA vs. 96.7% CLv2) and the lowest for insertion/deletion (indel) (88.4% OLcfDAN vs. 49.1% CLv2) confirmation. Detected allele frequencies of both panels showed the highest correlation for T790M mutations (98.5%) and the lowest for indels (63.4%). Tested hotspot mutations: *PIK3CA* E545K, *EGFR* V769_D770insASV, *EGFR* E746_A750delELREA, *EGFR* T790M, *EGFR* L858R, *KRAS* G12D, *NRAS* Q61K, *NRAS* A59T.

Correlation of expected reference allele frequencies and those derived from the CLv2 panel was 92% for point mutations and 97% specifically for T790M resistance mutation (Figures 1 and 2). Allele frequencies for indels showed a lower correlation of 49%. For the OLcfDNA panel, the correlation with the expected allele frequencies was 99% for point mutations and 100% for T790M resistance mutation, specifically. Correlation for indel detection was also lower, showing 88% accordance.

### Results from diagnostic samples

Primary *EGFR* mutations derived from the analysis of corresponding tumor tissue of the analyzed 26 LB from 21 patient samples were composed of 15 point mutations (11 patients), 10 indels (9 patients) and one unknown status (Supplementary Table 2).

**Figure 3:**
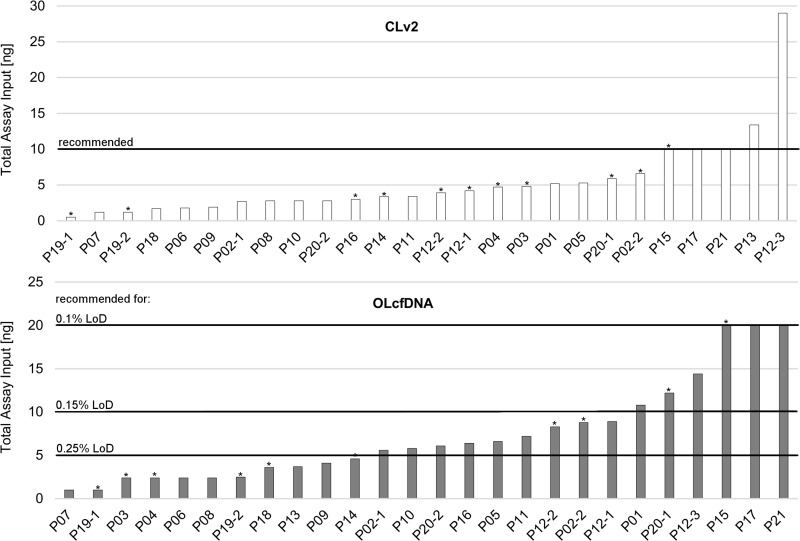
Achieved and recommended cfDNA input amount related to the limit of detection (LoD) for Colon Lung version 2 (CLv2) and Oncomine Lung cfDNA (OLcfDNA) assays; CLv2: 5/26 (19%) of clinical liquid biopsy (LB) samples yielded the recommended cfDNA amount for sequencing (10 ng) Median achieved assay input was 3.65 ng (range: 0.5 ng–29.0 ng). OLcfDNA: 3/26 (12%) samples fulfilled recommended specifications for 0.1% LoD. 6/26 (23%) yielded enough DNA for predetermined 0.15% LoD and 15/26 (58%) yielded the recommended amount for 0.25% LoD under terms of median read coverage > 25.000 and molecular coverage > 2.500. The median achieved assay input amount was 5.95 ng (range: 1.0 ng–20.0 ng). ^*^samples being wildtype or not analyzable by sequencing.

Median cfDNA concentration extracted from the 26 plasma samples was 8.88 ng/ml (range: 1.1 ng/ml–200 ng/ml plasma). The cfDNA input used for the panel amplification differed as optimal amount was not available for all samples (Figure 3). Five out of 26 (19%) plasma samples yielded enough cfDNA to comply to the recommended input requirements for NGS with CLv2 assay and 6/26 (23%) had sufficient cfDNA amounts for analysis with OLcfDNA approach for 0.1% LoD. The library concentrations ranged from 36pM to 8,151pM for CLv2 assay and from 22pM to 238pM for OLcfDNA assay. One sample failed library generation with the OLcfDNA panel and was thus not sequenced. For none of both assays a correlation of input amount and generated library concentration was detected.

Average reads per amplicon for CLv2 panel sequencing ranged from 1 to 27,605 (median: 5,621). OLcfDNA assay sequencing reached in 10/26 (39%) samples a median read coverage > 25,000 with 3 cases (12%) showing additionally a molecular coverage of > 2,500, required for 0.1% LoD.

**Figure 4:**
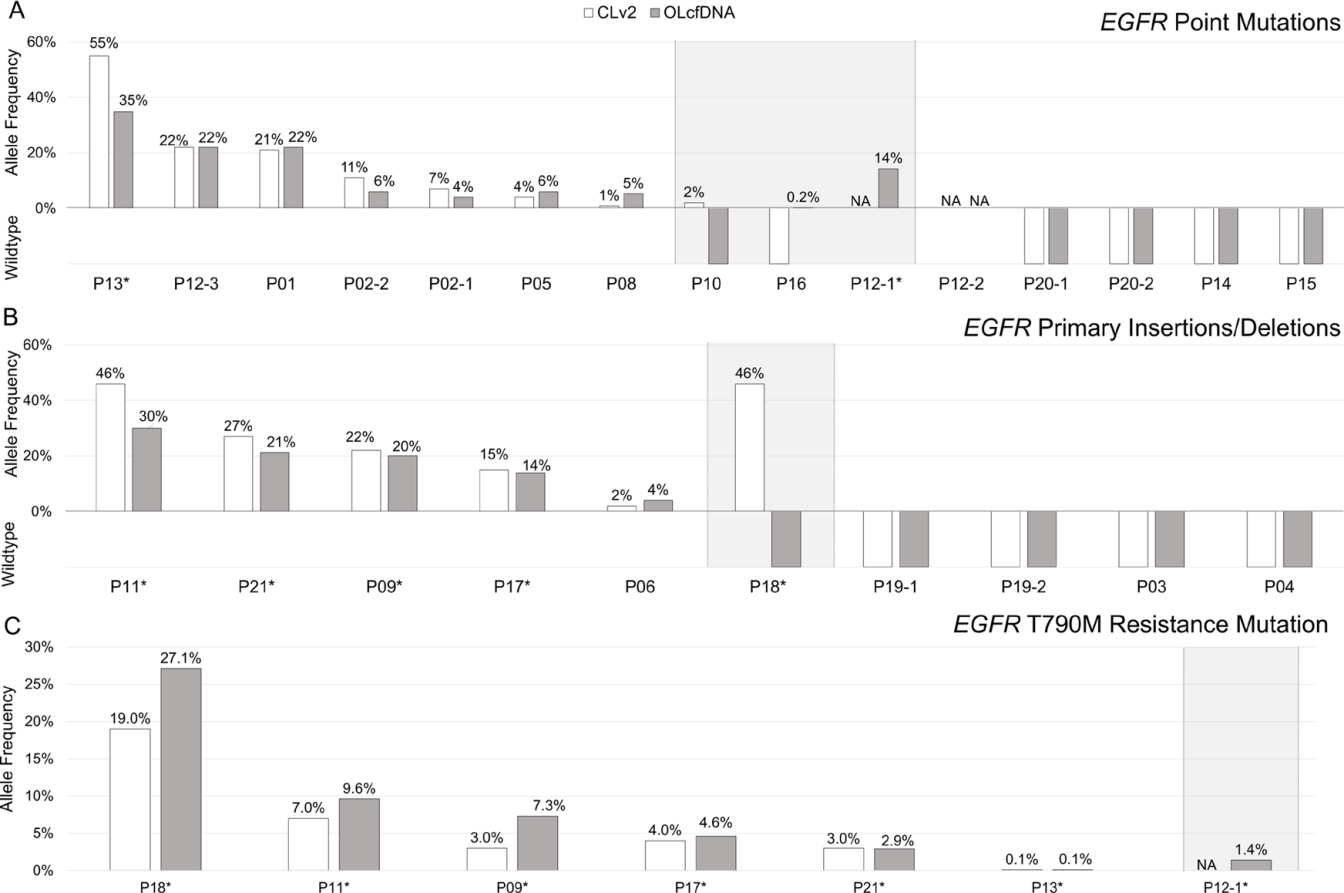
Comparison of Colon Lung version 2 (CLv2, white bars) and Oncomine Lung cfDNA (OLcfDNA, grey bars) mutation status of primary mutation positive non-small cell lung cancer (NSCLC) samples (**A**) CLv2 and OLcfDNA results for the 15 primary point mutations showed accordance in 12 cases (7 *EGFR* point mutation positive, 4 *EGFR* wildtype, 1 not analyzable (NA)). (**B**) Assays showed concordant results in 9/10 primary insertion/deletion mutated samples (5 insertion/deletion positive, 4 wildtype). One CLv2 positive sample was wildtype with OLcfDNA assay. (**C**) In six patients *EGFR* T790M mutation was detected with both assays with allele frequencies from 0.1% up to 27.1%. Another OLcfDNA *EGFR* T790M positive samples was not analyzable with CLv2 panel. ^*^EGFR primary and T790M mutation detected.

The assays showed concordant results in 12/15 cases with primary point mutations seven of which being primary mutation positive, four being wildtype and one being concordantly not analyzable (Figure 4A). For one sample (P10) the *EGFR* G863S primary point mutation was detected with 2% allele frequency and 21 reads with the CLv2 panel but was wildtype with the OLcfDNA assay. Another discordancy in sample P16 refers to a *EGFR* G719A primary mutation with 0.2% allele frequency and 53,214× median read coverage when being analyzed with the OLcfDNA assay and was wildtype with CLv2 panel. Analysis of primary indels led to 90% concordance between both assays (Figure 4B). Four out of the nine concordant cases were *EGFR* wildtype. The one discordant sample (P18) was mutation positive with CLv2 (46% allele frequency, 12,202 reads) and negative with OLcfDNA assay.

In six patients the *EGFR* T790M resistance mutation was detected with allele frequencies from 0.1% to 27% with both assays (Figure 4C). One sample (P12–1) was not analyzable with the CLv2 assay and but was T790M positive with the OLcfDNA assay showing an allele frequency of 1.4% and 40,160 median read coverage with 1,528 reads for molecular coverage. The primary mutation was detected in all samples showing the T790M mutation. Comparing data from both assays with results from primary mutation status, CLv2 and OLcfDNA showed similar sensitivity of 58.3%.

## DISCUSSION

With the European Medicines Agency (EMA) approval of a third generation TKI for advanced NSCLC patients, the request for *EGFR* T790M resistance mutation detection for stratification of lung cancer patients eligible for treatment increased significantly. Due to the advanced tumor stage of the respective NSCLC patients, the acquisition of tissue specimens may be difficult and the minimal-invasive analysis of LB is becoming increasingly attractive as an alternative approach to stratify patients according to their mutational profile. The use of cfDNA extracted from plasma of peripheral blood is currently the main source for this application [17, 21]. However, only a very small proportion of the cfDNA accounts for ctDNA reflecting the mutational profile of the tumor [22]. For that reason, methods allowing a very sensitive analysis are indispensable. Amplicon-based NGS methods enable a very sensitive mutation detection in addition to the analysis of a broad range of target genes. In our study, we evaluated two commercially available PCR-based NGS library preparation approaches, one including UMI, focusing on high sensitive *EGFR* T790M mutation detection in synthetic and diagnostic LB samples. Using a cfDNA spike-in dilution series from synthetic plasma we determined and compared the LoD, accuracy, specificity and sensitivity of both assays. Furthermore, we analyzed and compared the results of 26 diagnostic NSCLC blood samples subjected to sequencing with both assays. Although the two targeted panels were able to identify *EGFR* primary and T790M acquired resistance mutations, careful consideration of all tested reference dilutions and mutations demonstrates that the assay using UMI performed better in terms of LoD, accuracy, sensitivity and specificity - despite the fact that T790M mutation detection was feasible with both assays down to 0.1% LoD. In general, the detection of point mutations showed a better performance compared to indel identification, which is known to be challenging [23]. Beside the higher sensitivity of the UMI assay, the deviation in indel detection between both assays might be induced by different bioinformatics algorithms.

Our results of cfDNA isolation confirm recently published data concerning low DNA amounts extracted from plasma [24]. Although most of the diagnostic samples did not reach the recommended input amount only a few of these samples were either wildtype or not analyzable. In these samples the amount of ctDNA was probably too low to allow a sensitive detection of the T790M resistance or primary mutation. Interestingly, this was also the case in some LB samples with sufficient cfDNA input. These relations might be explained with a common theoretical example [25]. Calculating with 6.6pg DNA per cell, the input of 10 ng DNA corresponds to approximately 3,000 genomic copies. Using a sample with 100% tumor content, like our reference samples, 0.1% allele frequency mutation would mean to search for 3 mutated copies in 2,997 wildtype sequences. Thus, searching a 0.1% allele frequency mutation in 1% tumor content would mean to look for theoretically 0.03 copies. This is a remarkable low number and the *in silico* calculations don`t even consider the lower input amount, lower tumor content and the validated assay sensitivity < 100% in practice. Furthermore, the use of copies as cell equivalent seems to overestimate the calculated genomic copies. The cfDNA in the plasma is generated by apoptotic or necrotic processes and therefore the cell equivalent of the mutated gene copy is probably already degraded. These factors make input recommendations gratuitous and unpredictable. It explains why the *EGFR* mutation status seemed not to be linked to the recommended input amounts. It also explains the higher sensitivity of the OLcfDNA assay, which might be not only the use of UMI but the input of more DNA copies due to a higher input volume.

These data clearly illustrate that the main problem of LB analysis is the unavailability regarding the amount of the tumor DNA in the plasma samples. Dealing with an unknown amount of tumor-derived cfDNA makes diagnostic LB samples a black box and a validated or predetermined cut-off for LoD can therefore only be a vague reference. Only the implementation of tests allowing the exact determination of the tumor DNA content can make cut-off recommendations applicable. Until then, the absence of *EGFR* mutations in LB might not necessarily indicate that the tumor is indeed negative for these mutations and the complementary testing of a tissue biopsy is recommended [26]. Furthermore, there are additional factors that cannot be influenced and increase the complexity of LB analysis. The shedding of tumor-derived DNA into the blood stream is affected by several patient characteristics such as tumor size and stage, metastasis, inflammation, therapy status, fitness, and comorbidities [14, 22, 27, 28]. Additionally, pre-analytical factors such as blood tubes and cfDNA extraction methods play an important role for subsequent LB analysis. Finally, technical sensitivity cannot be expanded unlimited. Most DNA polymerases have an error rate of approximately 10^−5^ per basepair per cycle preventing reliable mutation detection below this threshold [29]. Currently, the parallel detection of (known) primary activating *EGFR* mutations and/or other tumor-specific mutations are helpful to ensure the presence of tumor-derived cfDNA in diagnostic LB samples. In our study, seven out of 21 diagnostic samples were T790M resistance positive and additionally showed the corresponding primary mutation. Nonetheless, some samples were only positive for primary mutation, indicating the presence of ctDNA and the real absence of the T790M mutation. It can be considered that these tumors follow a different resistance mechanism like MET amplification, constitutive IGF-1 activation or mutations in HER2, which cannot be detected with the employed assays [30].

We were able to detect *EGFR* T790M concordantly with both assays in 6/7 patients with one having the T790M resistance mutation at a very low allele frequency (0.1%). Another patient was exclusively T790M positive (1.4% allele frequency) with the more sensitive OLcfDNA assay. These results indicate that very high sensitivity is indeed needed for T790M mutation detection and that UMI-based gene panels have a better performance in some cases. This is important since even patients with a very low frequent T790M resistance mutation (< 10 copies/ml plasma) can benefit from treatment with of third generation TKIs [31].

In summary, a deep understanding of the entire LB workflow and highly standardized protocols are needed for the detection of *EGFR* T790M mutation and to ensure reliable and reproducible results for the benefit of a correct patient stratification. Targeted NGS approaches using UMI are able to improve sensitivity and specificity but further steps like determination of tumor DNA amount and panels allowing the detection of other resistance mechanism than T790M are needed to make LB an even more valuable tool for personalized medicine.

## MATERIALS AND METHODS

### Determination of assay performance using synthetic reference samples

DNA was isolated from synthetic plasma samples (Multiplex I cfDNA Reference Standard Set in synthetic plasma, Horizon Discovery, Waterbeach, UK) containing eight somatic hotspot mutations, including *EGFR* activating mutations and the T790M resistance mutation at different allelic frequencies (Supplementary Table 1). To this end, semi-automated magnetic bead-based extraction for large plasma volume was performed according to manufacturer's instruction (Maxwell RSC ccfDNA Plasma Kit, Custom, AX1114, Software version V1.0.1, Promega, Madison, WI, USA). cfDNA concentration was measured with a fluorescence assay (Quantus and QuantiFluor ONE dsDNA System, Promega). Subsequently, cfDNA from each sample was subjected to library preparation using Ion AmpliSeq Colon and Lung Cancer Research Panel v2 (CLv2, Thermo Fisher Scientific, Waltham, MA, USA) and Oncomine Lung cfDNA Assay (OLcfDNA, Thermo Fisher Scientific). For both panels the optimal amount of cfDNA input was used (CLv2: 10 ng in 6 μl, OLcfDNA: 20 ng in 13 μl).

CLv2 panel target amplification was performed by multiplex PCR using primer pairs for 92 amplicons covering hotspots of 22 genes.

The OLcfDNA assay consisted of 35 amplicons covering 169 mutation hotspots of 11 genes and enabled molecular tagging of each individual DNA input molecule using UMI. Sample processing for both panels was done according to the manufacturer's instructions (Ion AmpliSeq DNA and RNA Library Preparation, MAN0006735, Revision B.0 and Oncomine cfDNA Assays Part I: Library Preparation, MAN0014688, Revision E.0), using Ion Library TaqMan Quantitation Kit (Thermo Fisher Scientific) for library quantification and an Ion Chef system for template preparation. Finally, 30pM library pool containing eight samples per Ion 530 chip was sequenced on the Ion S5XL System (Thermo Fisher Scientific).

Assay performance was determined considering sensitivity, specificity, accuracy and LoD. To this end, CLv2 data (fastq files) were mapped to defined target regions and subjected to variant calling using Sequence Pilot software, version 4.3.1 build 502, module SeqNext (JSI Medical Systems, Ettenheim, Germany). Allele frequency cut-off for mutation detection, which is set to 5% for tissue sample analysis, was abrogated in this study in order to call the 1% and 0.1% allele frequencies of the reference samples. Therefore, data were filtered only according read quality score discarding reads with a quality score < 10 and amplicons having 40% of reads with a quality score < 10. OLcfDNA assay data (fastq files) analysis was performed employing Torrent Browser, Tagged Molecule Caller v.0.3.3. The application of UMI enables the provided variant caller cfDNA plug-in to group reads into molecular families. Random errors generated during the library construction and sequencing process are removed automatically. The Ion Reporter analysis uses at least three independent molecular families to identify and call a variant. To achieve 0.1% LoD (1 mutant copy in a background of 1,000 WT copies), 20 ng input DNA, > 25.000× median read coverage with > 2.500× median molecular coverage are recommended (https://www.thermofisher.com/order/catalog/product/A31149).

### Comparing assay performance using diagnostic samples

cfDNA from 26 liquid biopsy samples from 21 NSCLC patients (Supplementary Table 2) was extracted from BD Vacutainer EDTA Tubes (Becton Dickinson, Franklin Lakes, NJ, USA), PAXgene Blood ccfDNA Tubes (PreAnalytiX, Hombrechtikon, Switzerland) and Cell-Free RNA BCT Tubes (Streck, La Vista, NE, USA). For plasma preparation, whole-blood samples were centrifuged at 2,000× g for 10 minutes at 4°C. Plasma was transferred to 2 ml low-bind tubes and centrifuged at 17,949× g for 10 minutes at room temperature. Further, cfDNA isolation was done according to the same protocol used for the reference samples. If the cfDNA concentration was too low for the recommended assay input, the maximum input volume was used for library preparation. Mutation status was determined with both panels by sequencing employing an Ion S5XL sequencer (Thermo Fisher Scientific) as described above. Sequencing results were compared to mutation status from corresponding tissue collected either during in-house routine molecular diagnostics processing (Ion S5XL sequencing using CLv2 panel as described above) or by specification from external requestors. LB sequencing results determined with both panels were compared to each other according to the assay parameters ascertained with the reference standard.

## SUPPLEMENTARY MATERIALS AND TABLES







## Abbreviations

ARMS: amplification refractory mutation system
BEAMing: beads emulsion, amplification refractory mutation system
cfDNA: circulating cell-free DNA
CLv2: Ion AmpliSeq Colon and Lung Cancer Research Panel v2 (Thermo Fisher Scientific)
ctDNA: circulating cell-free tumor DNA
EGFR: epidermal growth factor receptor
LB: liquid biopsy
LoD: limit of detection
NGS: next generation sequencing
NSCLC: non-small cell lung cancer
OLcfDNA: Oncomine Lung cfDNA Assay (Thermo Fisher Scientific)
TKI: tyrosine kinase inhibitor
UMI: unique molecular identifier

## References

[1] Lynch TJ, Bell DW, Sordella R, Gurubhagavatula S, Okimoto RA, Brannigan BW, Harris PL, Haserlat SM, Supko JG, Haluska FG, Louis DN, Christiani DC, Settleman J (2004). Activating mutations in the epidermal growth factor receptor underlying responsiveness of non-small-cell lung cancer to gefitinib. N Engl J Med.

[2] Paez JG, Janne PA, Lee JC, Tracy S, Greulich H, Gabriel S, Herman P, Kaye FJ, Lindeman N, Boggon TJ, Naoki K, Sasaki H, Fujii Y (2004). EGFR mutations in lung cancer: correlation with clinical response to gefitinib therapy. Science.

[3] Mok TS, Wu YL, Thongprasert S, Yang CH, Chu DT, Saijo N, Sunpaweravong P, Han B, Margono B, Ichinose Y, Nishiwaki Y, Ohe Y, Yang JJ (2009). Gefitinib or carboplatin-paclitaxel in pulmonary adenocarcinoma. N Engl J Med.

[4] Rosell R, Carcereny E, Gervais R, Vergnenegre A, Massuti B, Felip E, Palmero R, Garcia-Gomez R, Pallares C, Sanchez JM, Porta R, Cobo M, Garrido P (2012). Erlotinib versus standard chemotherapy as first-line treatment for European patients with advanced EGFR mutation-positive non-small-cell lung cancer (EURTAC): a multicentre, open-label, randomised phase 3 trial. Lancet Oncol.

[5] Kobayashi S, Boggon TJ, Dayaram T, Janne PA, Kocher O, Meyerson M, Johnson BE, Eck MJ, Tenen DG, Halmos B (2005). EGFR mutation and resistance of non-small-cell lung cancer to gefitinib. N Engl J Med.

[6] Nguyen KS, Kobayashi S, Costa DB (2009). Acquired resistance to epidermal growth factor receptor tyrosine kinase inhibitors in non-small-cell lung cancers dependent on the epidermal growth factor receptor pathway. Clin Lung Cancer.

[7] Sequist LV, Waltman BA, Dias-Santagata D, Digumarthy S, Turke AB, Fidias P, Bergethon K, Shaw AT, Gettinger S, Cosper AK, Akhavanfard S, Heist RS, Temel J (2011). Genotypic and histological evolution of lung cancers acquiring resistance to EGFR inhibitors. Sci Transl Med.

[8] Janne PA, Yang JC, Kim DW, Planchard D, Ohe Y, Ramalingam SS, Ahn MJ, Kim SW, Su WC, Horn L, Haggstrom D, Felip E, Kim JH (2015). AZD9291 in EGFR inhibitor-resistant non-small-cell lung cancer. N Engl J Med.

[9] Cross DA, Ashton SE, Ghiorghiu S, Eberlein C, Nebhan CA, Spitzler PJ, Orme JP, Finlay MR, Ward RA, Mellor MJ, Hughes G, Rahi A, Jacobs VN (2014). AZD9291, an irreversible EGFR TKI, overcomes T790M-mediated resistance to EGFR inhibitors in lung cancer. Cancer Discov.

[10] Jiang T, Zhou C (2014). Clinical activity of the mutant-selective EGFR inhibitor AZD9291 in patients with EGFR inhibitor-resistant non-small cell lung cancer. Transl Lung Cancer Res.

[11] Chae YK, Davis AA, Carneiro BA, Chandra S, Mohindra N, Kalyan A, Kaplan J, Matsangou M, Pai S, Costa R, Jovanovic B, Cristofanilli M, Platanias LC, Giles FJ (2016). Concordance between genomic alterations assessed by next-generation sequencing in tumor tissue or circulating cell-free DNA. Oncotarget.

[12] Overman MJ, Modak J, Kopetz S, Murthy R, Yao JC, Hicks ME, Abbruzzese JL, Tam AL (2013). Use of research biopsies in clinical trials: are risks and benefits adequately discussed?. J Clin Oncol.

[13] Gormally E, Caboux E, Vineis P, Hainaut P (2007). Circulating free DNA in plasma or serum as biomarker of carcinogenesis: practical aspects and biological significance. Mutat Res.

[14] Forshew T, Murtaza M, Parkinson C, Gale D, Tsui DW, Kaper F, Dawson SJ, Piskorz AM, Jimenez-Linan M, Bentley D, Hadfield J, May AP, Caldas C (2012). Noninvasive identification and monitoring of cancer mutations by targeted deep sequencing of plasma DNA. Sci Transl Med.

[15] Diaz LA, Bardelli A (2014). Liquid biopsies: genotyping circulating tumor DNA. J Clin Oncol.

[16] Ye X, Zhu ZZ, Zhong L, Lu Y, Sun Y, Yin X, Yang Z, Zhu G, Ji Q (2013). High T790M detection rate in TKI-naive NSCLC with EGFR sensitive mutation: truth or artifact?. J Thorac Oncol.

[17] Xu S, Lou F, Wu Y, Sun DQ, Zhang JB, Chen W, Ye H, Liu JH, Wei S, Zhao MY, Wu WJ, Su XX, Shi R (2016). Circulating tumor DNA identified by targeted sequencing in advanced-stage non-small cell lung cancer patients. Cancer Lett.

[18] Cai X, Janku F, Zhan Q, Fan JB (2015). Accessing Genetic Information with Liquid Biopsies. Trends Genet.

[19] Crowley E, Di Nicolantonio F, Loupakis F, Bardelli A (2013). Liquid biopsy: monitoring cancer-genetics in the blood. Nat Rev Clin Oncol.

[20] Kivioja T, Vaharautio A, Karlsson K, Bonke M, Enge M, Linnarsson S, Taipale J (2011). Counting absolute numbers of molecules using unique molecular identifiers. Nat Methods.

[21] Lebofsky R, Decraene C, Bernard V, Kamal M, Blin A, Leroy Q, Rio Frio T, Pierron G, Callens C, Bieche I, Saliou A, Madic J, Rouleau E (2015). Circulating tumor DNA as a non-invasive substitute to metastasis biopsy for tumor genotyping and personalized medicine in a prospective trial across all tumor types. Mol Oncol.

[22] Diehl F, Schmidt K, Choti MA, Romans K, Goodman S, Li M, Thornton K, Agrawal N, Sokoll L, Szabo SA, Kinzler KW, Vogelstein B, Diaz LA (2008). Circulating mutant DNA to assess tumor dynamics. Nat Med.

[23] Ghoneim DH, Myers JR, Tuttle E, Paciorkowski AR (2014). Comparison of insertion/deletion calling algorithms on human next-generation sequencing data. BMC Res Notes.

[24] Bartels S, Persing S, Hasemeier B, Schipper E, Kreipe H, Lehmann U (2017). Molecular Analysis of Circulating Cell-Free DNA from Lung Cancer Patients in Routine Laboratory Practice: A Cross-Platform Comparison of Three Different Molecular Methods for Mutation Detection. J Mol Diagn.

[25] Volckmar AL, Sultmann H, Riediger A, Fioretos T, Schirmacher P, Endris V, Stenzinger A, Dietz S (2018). A field guide for cancer diagnostics using cell-free DNA: From principles to practice and clinical applications. Genes Chromosomes Cancer.

[26] Oxnard GR, Thress KS, Alden RS, Lawrance R, Paweletz CP, Cantarini M, Yang JC, Barrett JC, Janne PA (2016). Association Between Plasma Genotyping and Outcomes of Treatment With Osimertinib (AZD9291) in Advanced Non-Small-Cell Lung Cancer. J Clin Oncol.

[27] Tug S, Helmig S, Deichmann ER, Schmeier-Jurchott A, Wagner E, Zimmermann T, Radsak M, Giacca M, Simon P (2015). Exercise-induced increases in cell free DNA in human plasma originate predominantly from cells of the haematopoietic lineage. Exerc Immunol Rev.

[28] Bettegowda C, Sausen M, Leary RJ, Kinde I, Wang Y, Agrawal N, Bartlett BR, Wang H, Luber B, Alani RM, Antonarakis ES, Azad NS, Bardelli A (2014). Detection of circulating tumor DNA in early- and late-stage human malignancies. Sci Transl Med.

[29] Li M, Diehl F, Dressman D, Vogelstein B, Kinzler KW (2006). BEAMing up for detection and quantification of rare sequence variants. Nat Methods.

[30] Lin Y, Wang X, Jin H (2014). EGFR-TKI resistance in NSCLC patients: mechanisms and strategies. Am J Cancer Res.

[31] Buder A, Hochmair M, Holzer S, Mohn-Staudner A, Errhalt P, Bundalo T, Schenk P, Setinek U, Burghuber O, Pirker R, Filipits M, Adjei AA (2017). P3.02b-032 Association between EGFR T790M Mutation Copy Numbers in Cell-Free Plasma DNA and Response to Osimertinib in Advanced NSCLC.

