# Supplementary material for: Validation and comparison of two NGS assays for the detection of EGFR T790M resistance mutation in liquid biopsies of NSCLC patients
Source: Oncotarget. 2018 Apr 6;9(26):18529–39. doi: 10.18632/oncotarget.24908 (PMC5915090; doi:10.18632/oncotarget.24908)
Supplement: Supplementary file 1 [file oncotarget-09-18529-s001.pdf]

# Validation and comparison of two NGS assays for the detection of EGFR T790M resistance mutation in liquid biopsies of NSCLC patients

## SUPPLEMENTARY MATERIALS

**Supplementary Table 1: Somatic variants of the multiplex reference standard and expected allele frequencies**

| | | Expected allelic frequency (%) | | |
|---|---|---|---|---|
| **Gene** | **Variant** | **0.1% reference standard** | **1% reference standard** | **5% reference standard** |
| *EGFR* | L858R | 0.10 | 1.00 | 5.00 |
| *EGFR* | ΔE746-A750 | 0.10 | 1.00 | 5.00 |
| *EGFR* | T790M | 0.10 | 1.00 | 5.00 |
| *EGFR* | V769_D770insASV | 0.10 | 1.00 | 5.00 |
| *KRAS* | G12D | 0.13 | 1.30 | 6.30 |
| *NRAS* | Q61K | 0.13 | 1.30 | 6.30 |
| *NRAS* | A59T | 0.13 | 1.30 | 6.30 |
| *PIK3CA* | E545K | 0.13 | 1.30 | 6.30 |

**Supplementary Table 2: Clinical non-small cell lung cancer (NSCLC) samples analyzed in this study.** See_Supplementary_Table 2

**Supplementary Table 3: Reference samples (synthetic plasma) analyzed in this study.** See_ Supplementary_Table 3
