# Supplementary material for: Validation and comparison of two NGS assays for the detection of EGFR T790M resistance mutation in liquid biopsies of NSCLC patients
Source: Oncotarget. 2018 Apr 6;9(26):18529–39. doi: 10.18632/oncotarget.24908 (PMC5915090; doi:10.18632/oncotarget.24908)
Supplement: Supplementary file 2 [file oncotarget-09-18529-s002.docx]

Supplementary Table 2: Clinical non-small cell lung cancer (NSCLC) samples analyzed in this study; WT: wildtype, NA: Not Analyzable

|  |  |  | **Assay Input [ng]** | | **Library [pM]** | | **Coverage** | | |  | ***EGFR* Primary Mutation** | | | | | ***EGFR* T790M Resistance Mutation** | | | | |
| --- | --- | --- | --- | --- | --- | --- | --- | --- | --- | --- | --- | --- | --- | --- | --- | --- | --- | --- | --- | --- |
| **Patient** | **Blood Collection Tube** | **cfDNA ng/ml Plasma** | **CLv2** | **OLcfDNA** | **CLv2** | **OLcfDNA** | **CLv2 (Average Reads per Amplicon)** | **OLcfDNA (Median Read Coverage)** | **OLcfDNA (Molecular Coverage)** | **Amino Acid Change Primary Mutation** | **CLv2 Allele Frequency** | **CLv2 Coverage** | **OLcfDNA Allele Frequency** | **OLcfDNA (Median Read Coverage)** | **OLcfDNA (Molecular Coverage)** | **CLv2 Allele Frequency** | **CLv2 Coverage** | **OLcfDNA Allele Frequency** | **OLcfDNA (Median Read Coverage)** | **OLcfDNA (Molecul Coverage)** |
| P01 | EDTA | 13.58 | 5.2 | 10.8 | 4,764 | 93 | 8,633 | 21,896 | 1,612 | L861Q | 21.0% | 1,324 | 22.0% | 42,460 | 1,553 | WT | | WT | | |
| P02-1 | EDTA | 6.68 | 2.7 | 5.6 | 2,184 | 73 | 10,841 | 40,200 | 1,037 | L861Q | 9.0% | 586 | 4.0% | 76,149 | 941 | WT | | WT | | |
| P02-2 | EDTA | 11.00 | 6.6 | 8.8 | 4,164 | 74 | 7,890 | 44,319 | 1,357 | L861Q | 11.0% | 599 | 6.0% | 83,915 | 1,257 | WT | | WT | | |
| P03 | Streck | 9.75 | 4.8 | 2.4 | 499 | 35 | 4,305 | 7,842 | 131 | E746_A750delELREA | WT | | WT | | | WT | | WT | | |
| P04 | Streck | 11.27 | 4.7 | 2.4 | 212 | 33 | 3,913 | 15,173 | 222 | Ex19del | WT | | WT | | | WT | | WT | | |
| P05 | Streck | 9.21 | 5.3 | 6.6 | 584 | 53 | 772 | 37,608 | 816 | L858R | 4.0% | 29 | 6.0% | 51,096 | 615 | WT | | WT | | |
| P06 | Streck | 2.03 | 1.8 | 2.4 | 365 | 32 | 4,301 | 19,383 | 363 | E746_A750delELREA | 2.0% | 79 | 4.0% | 21,638 | 417 | WT | | WT | | |
| P07 | Streck | 4.47 | 1.2 | 1.0 | 223 | 43 | 4,089 | 3,812 | 61 | unknown | WT | | WT | | | WT | | WT | | |
| P08 | Streck | 5.61 | 2.8 | 2.4 | 331 | 38 | 20,077 | 11,955 | 163 | L858R | 0.8% | 25 | 5.2% | 19,268 | 136 | WT | | WT | | |
| P09 | Streck | 4.28 | 1.9 | 4.1 | 152 | 50 | 4,745 | 23,663 | 512 | E746_A750delELREA | 22.0% | 1,313 | 20.1% | 32,849 | 726 | 3.0% | 84 | 7.3% | 22,486 | 480 |
| P10 | Streck | 3.07 | 2.8 | 5.8 | 43 | 30 | 15,117 | 7,193 | 99 | G863S | 2.0% | 21 | WT | | | WT | | WT | | |
| P11 | Streck | 8.55 | 3.4 | 7.2 | 1,558 | 79 | 9,802 | 47,227 | 1,255 | E746_A750delELREA | 46.0% | 2,425 | 30.1% | 68,600 | 1,974 | 7.00% | 480 | 9.6% | 41,498 | 1,151 |
| P12-1 | Streck | 18.37 | 4.2 | 8.9 | 75 | 111 | 0 | 42,585 | 1,613 | L858R | NA | | 14.3% | 75,216 | 1,514 | NA | | 1.4% | 40,160 | 1,528 |
| P12-2 | PAX | 17.11 | 3.9 | 8.3 | 36 | 0 | 6 | - | - | L858R | NA | | NA | | | NA | | NA | | |
| P-12-3 | Streck | 85.16 | 29.0 | 14.4 | 8,151 | 98 | 25,353 | 60,896 | 2,446 | L858R | 22,0% | 3,056 | 22,0% | 151,054 | 2,426 | WT | | WT | | |
| P13 | PAX | 29.38 | 13.4 | 3.7 | 3,943 | 101 | 27,605 | 91,982 | 1,272 | L858R | 55.0% | 11,775 | 34.8% | 167,658 | 1,468 | 0.1% | 30,692 | 0.1% | 107,726 | 1,507 |
| P14 | Streck | 34.83 | 3.4 | 4.6 | 99 | 119 | 16,571 | 7,493 | 109 | G719A | WT | | WT | | | WT | | WT | | |
| P15 | PAX | 200.00 | 10.0 | 20.0 | 5,049 | 170 | 10,510 | 144,760 | 2,758 | L858R | WT | | WT | | | WT | | WT | | |
| P16 | Streck | 7.20 | 3.0 | 6.4 | 50 | 238 | 1 | 27,838 | 578 | G719A | WT | | WT | | | WT | | WT | | |
| P17 | PAX | 67.00 | 10.0 | 20.0 | 1,265 | 176 | 22,666 | 22,043 | 2,593 | E746_A750delELREA | 15.0% | 3,818 | 13.8% | 30,079 | 3,058 | 4.0% | 659 | 4.6% | 15,824 | 1,667 |
| P18 | Streck | 7.20 | 1.7 | 3.6 | 1,341 | 80 | 19,856 | 8,146 | 481 | T751_I759delinsN | 46.0% | 12,202 | WT | | | 19.0% | 3,278 | 27.1% | 7,153 | 462 |
| P19-1 | PAX | 1.10 | 0.5 | 1.0 | 335 | 59 | 9,329 | 4,322 | 187 | E746_S752delinsV | WT | | WT | | | WT | | WT | | |
| P19-2 | Streck | 2.00 | 1.2 | 2.5 | 736 | 88 | 8,950 | 5,531 | 267 | E746_S752delinsV | WT | | WT | | | WT | | WT | | |
| P20-1 | Streck | 8.08 | 5.9 | 12.2 | 485 | 106 | 12,730 | 6,769 | 577 | L858R | WT | | WT | | | WT | | WT | | |
| P20-2 | PAX | 3.84 | 2.8 | 6.1 | 851 | 64 | 13,631 | 7,938 | 461 | L858R | WT | | WT | | | WT | | WT | | |
| P21 | Streck | 57.50 | 10.0 | 20.0 | 1,582 | 22 | 26,637 | 258,403 | 4,510 | E746_A750delELREA | 27.0% | 9,656 | 21.2% | 399,025 | 5,927 | 3.0% | 516 | 2.9% | 233,077 | 3,621 |
