# Supplementary material for: Validation and comparison of two NGS assays for the detection of EGFR T790M resistance mutation in liquid biopsies of NSCLC patients
Source: Oncotarget. 2018 Apr 6;9(26):18529–39. doi: 10.18632/oncotarget.24908 (PMC5915090; doi:10.18632/oncotarget.24908)
Supplement: Supplementary file 3 [file oncotarget-09-18529-s003.docx]

Supplementary Table 3: Reference samples (synthetic plasma) analyzed in this study; ND: Not Detected

|  |  |  | **Assay Input [ng]** | | **Library [pM]** | | **Coverage** | | |  |  | | | | |
| --- | --- | --- | --- | --- | --- | --- | --- | --- | --- | --- | --- | --- | --- | --- | --- |
| **Reference** | **Blood Collection Tube** | **cfDNA ng/ml Plasma** | **CLv2** | **OLcfDNA** | **CLv2** | **OLcfDNA** | **CLv2 (Average Reads per Amplicon)** | **OLcfDNA (Median Read Coverage)** | **OLcfDNA (Molecular Coverage)** | ***EGFR* Mutation** | **CLv2 Allele Frequency** | **CLv2 Coverage** | **OLcfDNA Allele Frequency** | **OLcfDNA (Median Read Coverage)** | **OLcfDNA (Molecular Coverage)** |
| WT | - | 182.00 | 10.0 | 20.0 | 1,482 | 182 | 20,656 | 53,064 | 2,553 | c.2307_2308insGCCAGCGTG, p.V769_D770insASV | ND | ND | ND | ND | ND |
|  |  |  |  |  |  |  |  |  |  | c.2236_2250del15, p.E746_A750delELREA | ND | ND | ND | ND | ND |
|  |  |  |  |  |  |  |  |  |  | c.2369C>T, p.T790M | ND | ND | ND | ND | ND |
|  |  |  |  |  |  |  |  |  |  | c.2573T>G, p.L858R | ND | ND | ND | ND | ND |
| WT | - | 220.00 | 10.0 | 2.0 | 992 | 233 | 18,851 | 51,711 | 2,502 | c.2307_2308insGCCAGCGTG, p.V769_D770insASV | ND | ND | ND | ND | ND |
|  |  |  |  |  |  |  |  |  |  | c.2236_2250del15, p.E746_A750delELREA | ND | ND | ND | ND | ND |
|  |  |  |  |  |  |  |  |  |  | c.2369C>T, p.T790M | ND | ND | ND | ND | ND |
|  |  |  |  |  |  |  |  |  |  | c.2573T>G, p.L858R | ND | ND | ND | ND | ND |
| 0.1% | - | 240.00 | 10.0 | 20.0 | 963 | 206 | 19,302 | 58,422 | 2,862 | c.2307_2308insGCCAGCGTG, p.V769_D770insASV | ND | ND | 0.1% | 44,003 | 2,985 |
|  |  |  |  |  |  |  |  |  |  | c.2236_2250del15, p.E746_A750delELREA | ND | ND | 0.3% | 68,360 | 3,083 |
|  |  |  |  |  |  |  |  |  |  | c.2369C>T, p.T790M | 0.0% | 21,092 | 0.1% | 64,837 | 3,033 |
|  |  |  |  |  |  |  |  |  |  | c.2573T>G, p.L858R | ND | ND | 0.3% | 67,509 | 1,570 |
| 0.1% | - | 220.00 | 10.0 | 20.0 | 1,482 | 167 | 23,195 | 58,498 | 2,964 | c.2307_2308insGCCAGCGTG, p.V769_D770insASV | ND | ND | ND | ND | ND |
|  |  |  |  |  |  |  |  |  |  | c.2236_2250del15, p.E746_A750delELREA | ND | ND | 0.1% | 66,927 | 3,158 |
|  |  |  |  |  |  |  |  |  |  | c.2369C>T, p.T790M | 0.0% | 20,592 | 0.1% | 61,998 | 3,150 |
|  |  |  |  |  |  |  |  |  |  | c.2573T>G, p.L858R | ND | ND | ND | ND | ND |
| 1% | - | 200.00 | 10.0 | 20.0 | 1,181 | 141 | 23,046 | 30,958 | 2,482 | c.2307_2308insGCCAGCGTG, p.V769_D770insASV | ND | ND | 0.6% | 21,990 | 2,496 |
|  |  |  |  |  |  |  |  |  |  | c.2236_2250del15, p.E746_A750delELREA | ND | ND | 1.7% | 36,344 | 2,759 |
|  |  |  |  |  |  |  |  |  |  | c.2369C>T, p.T790M | 0.3% | 19,946 | 0.9% | 34,349 | 2,733 |
|  |  |  |  |  |  |  |  |  |  | c.2573T>G, p.L858R | 0.0% | 14,874 | 1.1% | 37,251 | 1,372 |
| 1% | - | 240.00 | 10.0 | 20.0 | 944 | 214 | 24,438 | 47,100 | 2,635 | c.2307_2308insGCCAGCGTG, p.V769_D770insASV | 0.1% | 24,951 | 0.6% | 36,368 | 2,823 |
|  |  |  |  |  |  |  |  |  |  | c.2236_2250del15, p.E746_A750delELREA | 0.4% | 22,996 | 1.5% | 59,289 | 2,971 |
|  |  |  |  |  |  |  |  |  |  | c.2369C>T, p.T790M | 0.7% | 24,715 | 0.8% | 55,318 | 2,972 |
|  |  |  |  |  |  |  |  |  |  | c.2573T>G, p.L858R | 0.4% | 19,161 | 0.7% | 55,591 | 1,500 |
| 5% | - | 220.00 | 10.0 | 20.0 | 1,071 | 183 | 17,514 | 47,739 | 2,747 | c.2307_2308insGCCAGCGTG, p.V769_D770insASV | 4.0% | 17,655 | 4.1% | 41,068 | 2,955 |
|  |  |  |  |  |  |  |  |  |  | c.2236_2250del15, p.E746_A750delELREA | 8.0% | 18,243 | 7.2% | 57,164 | 2,980 |
|  |  |  |  |  |  |  |  |  |  | c.2369C>T, p.T790M | 4.0% | 17,601 | 4.8% | 54,658 | 2,874 |
|  |  |  |  |  |  |  |  |  |  | c.2573T>G, p.L858R | 2.6% | 15,363 | 5.2% | 48,020 | 1,449 |
| 5% | - | 220.00 | 10.0 | 20.0 | 374 | 228 | 22,062 | 45,347 | 2,672 | c.2307_2308insGCCAGCGTG, p.V769_D770insASV | 0.1% | 23,616 | 3.6% | 35,859 | 2,914 |
|  |  |  |  |  |  |  |  |  |  | c.2236_2250del15, p.E746_A750delELREA | 1.9% | 21,452 | 5.2% | 56,241 | 3,092 |
|  |  |  |  |  |  |  |  |  |  | c.2369C>T, p.T790M | 5.2% | 22,878 | 5.3% | 46,396 | 2,891 |
|  |  |  |  |  |  |  |  |  |  | c.2573T>G, p.L858R | 2.4% | 17,100 | 6.1% | 54,342 | 1,486 |
